# Health Tract, No. 182

**Published:** 1863-12

**Authors:** 


### MEDICAL MELANGE.

Blistered Hands and Feet.—The speediest remedy is to light a tallow candle and let the melted tallow drop in cold water, then mix the tallow with strong spirits and rub it thoroughly into the palms or soles ; this is both a preventive and curative.

Concentrated Potatoes.—A bushel of potatoes averages sixty pounds ; when all the water is absorbed five pounds of nutritive material are left, which, when ground, looks like Indian (corn) meal. A factory in Maine "concentrates" a thousand bushels of potatoes a day for the army.

Reading whilst Traveling fatigues the eyes, as every observant person well knows ; this induces headache, sometimes pains around the eyes, with a slight congestion of the retina, which, when the habit becomes inveterate, and the subject is over fifty or of a weak constitution, is liable to end in an attack of apoplexy. London medical journals have reported several very obscure, painful, and complex maladies which have entirely disappeared, and very promptly too, on the discontinuance of the custom of reading on rail-cars while in motion or of riding long distances to business daily, namely, thirty or forty miles every day.

Poisoned Ivy or Oak-Vines.—Some persons are so susceptible of being poisoned in passing through the woods, that a breath of air passing from the vine toward any exposed part of the body is sufficient to produce severe skin disease, and which is very difficult of cure in some constitutions. An item has been going the round of the papers lately to the effect that if the person will chew and swallow even half a leaf of the ivy itself it effects a speedy cure. But the public should be on their guard in using this remedy, for one case at least is given where swallowing a single leaf was followed by most distressing symptoms, and the person barely escaped with life.

Hydrophobia is said to be cured promptly and effectually by swallowing a decoction of thorn-apple, that is the plant known as the Jamestown or Jimson weed, putting the patient in a furious rage as soon as swallowed. When it is known that this weed is a deadly poison under ordinary circumstances, and that children have frequently died after having eaten a few seeds, persons are counseled not to make the experiment, and to let poison-oak and the thorn-apple alone ; it is always better to apply to a physician.

Taking Colds.—Some persons can almost tell in an instant when they have taken cold, generally by the disagreeable feeling of chilliness and the difficulty of getting comfortably warmed. Sometimes a person after exercising actively finds himself a little chilled before he knows it. In both cases an available, instantaneous, and almost always efficient remedy is at hand—simply walk, run, or work until free perspiration is produced, the sooner the better, and when the exercise is over, go to a room of seventy degrees Fahrenheit, or drink several cups of hot drink, taking care, if not in a warm room, to cease exercising by degrees.

Impressions on the Retina after Death.—This is a beautiful thought ; too beautiful to be true. Impressions do not remain an instant in life, for another one comes as fast as presented. How, then, can they remain after death ? The last impression in life does remain some time, if the eye is immediately closed, as Sir Isaac Newton describes in his own experience, in a letter to the philosopher Locke ; but to do that it was necessary that he should fix the mind on it, or as Sir Isaac expressed himself, "intend my fancy," but such an *intending* can not exist after death.

Diphtheria is said to be speedily arrested and cured by swallowing lumps of ice, *continuously*, until relief is afforded ; let them as much as possible melt in the throat. Common sore-throat is cured in the same way, sometimes.

Epilepsy, or falling sickness, is reported to be successfully treated by Dr. John Chapman, of London, editor of the *Westminster Review*, by the application of ice and hot water, in India-rubber bags, at various parts of the spinal column.

